# Follicular dendritic cell sarcoma (FDCS) of urinary bladder with coexisting urothelial carcinoma–a case report

**DOI:** 10.1186/s12894-019-0517-x

**Published:** 2019-09-05

**Authors:** Jing Sun, Cheng Wang, Dandan Wang, Jiangtao Wu, Leiming Wang, Lan Zhao, Lianghong Teng

**Affiliations:** 1 Department of Pathology, Xuan Wu Hospital, Capital Medical University, Beijing, China; 2 Department of Pathology, Capital Medical University, Beijing, China; 3 Department of Urology, Xuan Wu Hospital, Capital Medical University, Beijing, China; 4 Department of Pathology and Laboratory Medicine, Dalhousie University, Nova Scotia, Canada

**Keywords:** Follicular dendritic cell sarcoma, Urothelial carcinoma, Urinary bladder, Transurethral resection of bladder tumor, Coexisting

## Abstract

**Background:**

Follicular dendritic cell sarcoma is a very rare bladder tumor with very few cases that have been reported in the English literature.

**Case presentation:**

We report an unusual case of follicular dendritic cell sarcoma that is coexistent with urothelial carcinoma (UC) in the urinary bladder of a 73-year-old man, who first presented with lower abdominal pain. Microscopic examination of the first transurethral resection of bladder tumor (TURBT) sample showed a neoplasm containing spindle or ovoid-shaped cells that were arranged in storiform, nested or swirling patterns. Abundant mitotic Figs. (30 mitoses/10 high-power fields) and apoptotic bodies were present. The tumor cells were positive for CD21 and vimentin, partly positive for CD23, D2–40 and CD35. After 6 weeks, the tumor recurred lately, which surprisingly contained a component of urothelial carcinoma. The first TURBT sample was then reviewed and a coexisting UC mixed with FDCS was identified by examining the deeper levels of the tumor blocks.

**Conclusions:**

This case is, to our knowledge, the first time to report the coexistence of FDCS and UC in the urinary bladder of an elderly patient. And these two tumors may share a similar molecular mechanism.

## Background

Follicular dendritic cell sarcoma (FDCS) is a rare neoplasm involving the proliferation of neoplastic dendritic cells [1, 2]. When it occurs in extra nodal sites, it is well known to be a diagnostic challenge [3, 4]. To our knowledge, up till now only one case of FDCS has been reported in urinary bladder [5]. There are only rare reports that FDCS can occur concurrently with other types of tumors such as Castleman disease [3]. Here we report a challenging case of FDCS in the urinary bladder, particularly coexisted with urothelial carcinoma (UC).

## Case presentation

**Fig. 1:**
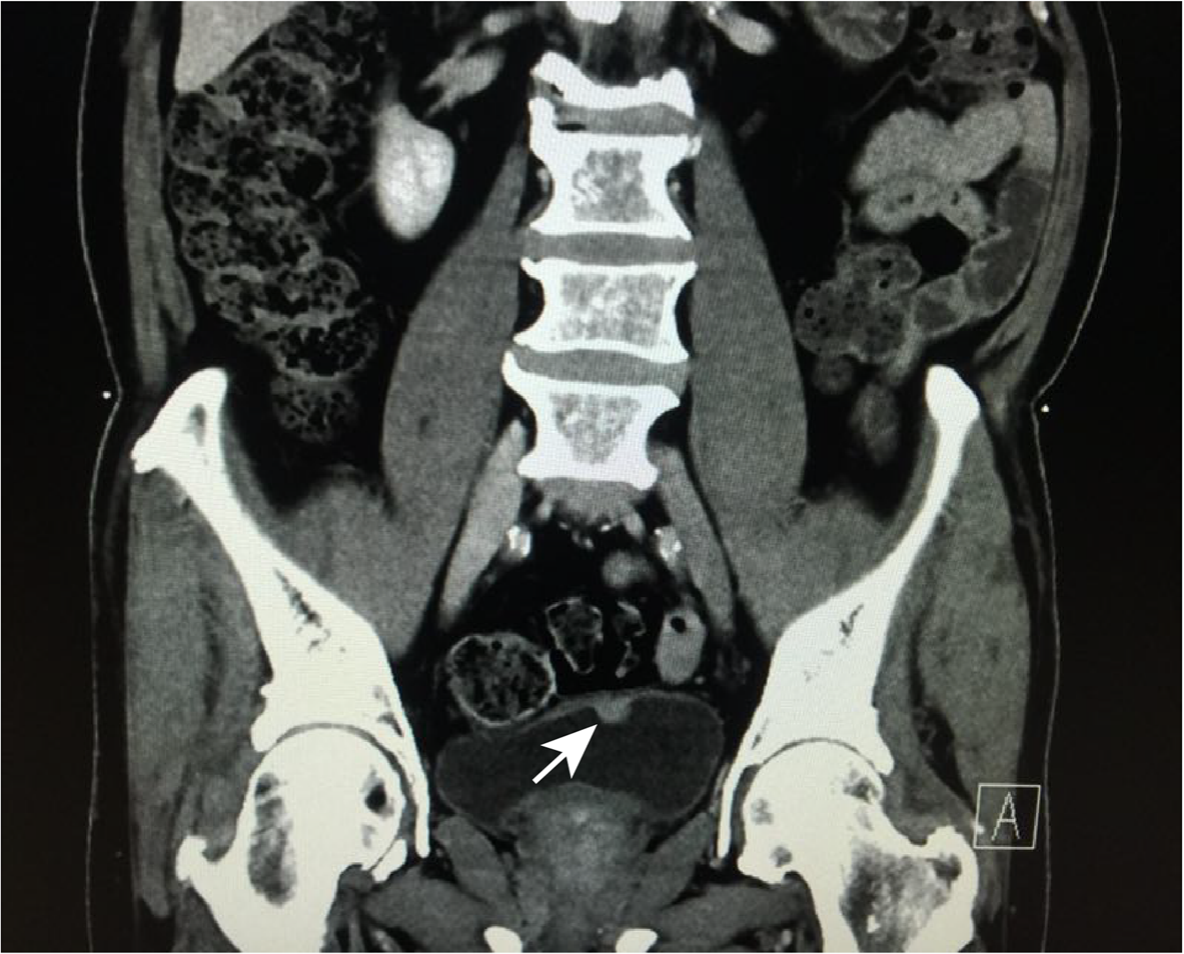
Pelvic computed tomography examination shows a nodular soft tissue shadow (arrow) at the left anterior wall of the bladder

**Fig. 2:**
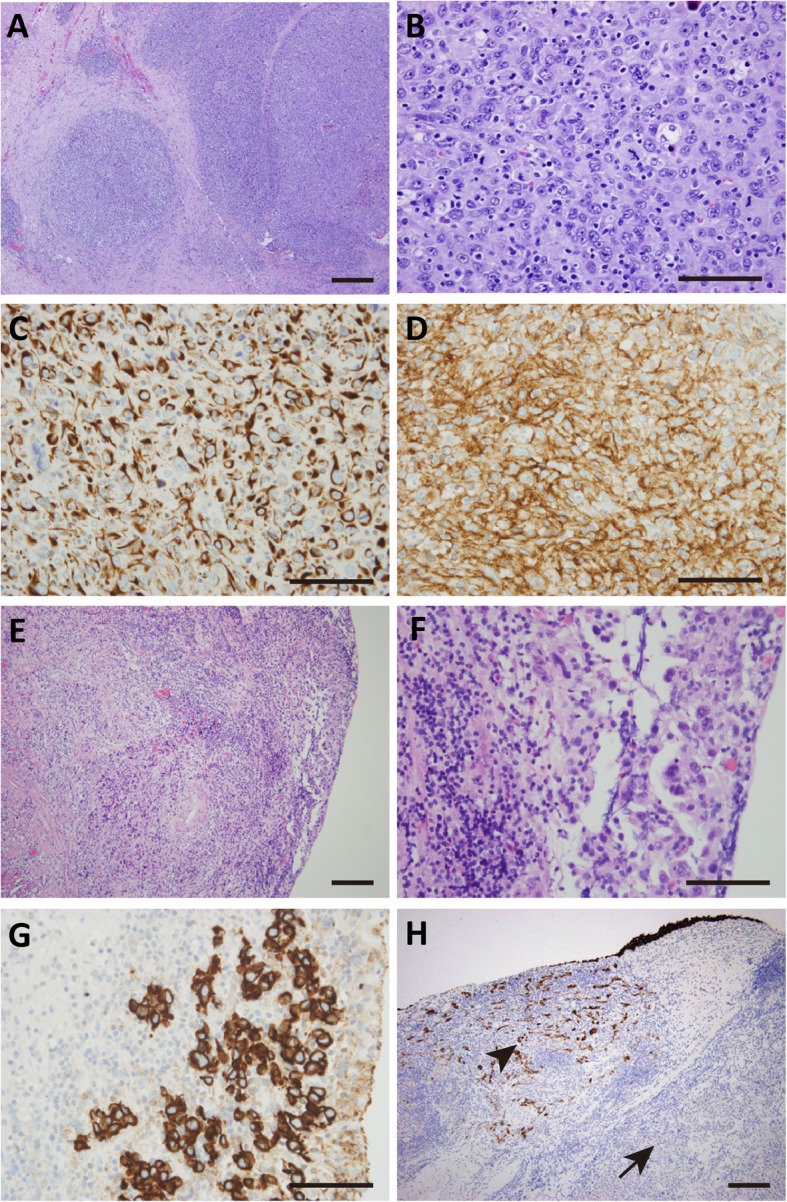
HE and immunohistochemistry staining of the bladder tumor. HE staining **a**-**b**) showed FDCS tumor cells arranged in a storiform or nesting pattern, tumor cells had indistinct cytoplasmic borders, round or ovoid nuclei; Immunohistochemistry staining showed FDCS tumor cells were positive for vimentin (**c**) and CD21 (**d**); HE staining (**e**-**f**) showed UC tumor cells arranged in nest or cord pattern, tumor cells had acidophilic cytoplasm and irregular nuclear; Immunohistochemistry staining showed UC tumor cells were positive for CK (**g**); Immunohistochemistry staining of CK (**h**) showed infiltrated UC (arrow head) mixed with FDCS (arrow), UC were positive for CK, FDCS were negative for CK. Bar = 200 μm

In August 2016, a cystoscopically visible protuberant neoplasm of the urinary bladder was found in a 73-year-old man, with clinical manifestation of lower abdominal pain, frequency, urgency and dysuria during urination. Pelvic computed tomography (CT) examination showed a 1.5 cm nodular soft tissue shadow at the left anterior wall of the bladder (Fig. 1). The patient then underwent the procedure of transurethral resection of bladder tumor (TURBT). Resected sample was formalin fixed, paraffin embedded. The tissue blocks were cut into 3-μm sections, which were stained with hematoxylin and eosin. Microscopic examination showed the neoplasm was composed of spindle or ovoid-shaped cells that formed storiform, nested or swirling patterns. It involved mucosa and submucosa layers. The neoplastic spindle cells had indistinct cytoplasmic borders, a moderate amount of lightly acidophilic cytoplasm, round or ovoid nuclei with a thin nuclear membrane and small nucleoli. Abundant mitotic Figs. (30 mitoses/10 high-power fields) and apoptotic bodies were present, with no necrosis and hemorrhage. Multinucleated cells and pleomorphic cells were also seen. Some mature lymphocytes infiltrated between tumor cells and in perivascular spaces (Fig. 2a, b). The residual lymphoid tissue was limited to small follicles.

Immunohistochemical stains were performed in our laboratory, utilizing an avidin biotin peroxidase complex method. Heat-induced antigen retrieval was performed and then the tissue was incubated with antibodies. Mouse monoclonal anti-human antibodies against CD3, CD5, CD20, CD21, CD23, CD30, CD56, CK, CK7, EMA, HMB45, Melan A, SMA, Vimentin, rabbit polyclonal anti-human antibodies against S-100, were purchased from Leica company. Mouse monoclonal anti-human antibodies CD35, D2–40, Desmin, Ki-67, MPO, P63, GATA-3, P16, P53, EGFR, ALK, CK5/6, rabbit polyclonal anti-human antibodies against CK20, P40, TFE-3, Uroplakin, were purchased from ZS company. Mouse monoclonal anti-human antibody BRAF V600E (VE1) was purchased from Roche company.

The tumor cells were positive for CD21 and vimentin, partly positive for CD23, D2–40 and CD35. The tumor cells were negative for CK, CK5/6, EMA, CK7, CK20, P63, P40, Uroplakin, Desmin, SMA, S100, TFE-3, HMB45, MelanA, MPO, ALK, CD3, CD5, CD20 and CD30. Ki-67 was expressed in about 30% of the tumor cell nuclei (Fig. 2c, d). Silver staining demonstrated abundant fibers circumfused each tumor cell. The pathological diagnosis of follicular dendritic cell sarcoma was given based on the morphology and immunohistochemistry.

Six weeks later, the tumor recurred, which appeared widely based, deeper than the primary surgical scar and was about 1.5 × 2 cm in size. A second transurethral resection was performed and microscopically the FDCS still could be seen in bladder mucosa and submucosa. FDCS tumor cells were similar to those seen in the previous sample, which were spindle-shaped with round or ovoid nuclei with small nucleoli. But the number of mitotic Figs. (10 mitoses/10 high-power fields) was lower than that of the first sample. However, the tumor cells were found to infiltrate in muscularis propria. It was surprising that there was also an invasive urothelial carcinoma that was mixed with the FDCS. The UC of bladder infiltrated in mucosa and submucosa. The tumor cells of UC were arranged in nest or cord pattern, the cytoplasm was acidophilic and the nuclear were irregular. (Fig. 2e, f). Using immunohischemistry, UC were positive for CK, CK20, P63, GATA-3, negative for CD21, CD23, CD35 and D2–40. Otherwise, FDCS were positive for Vimentin, CD21, CD23, CD35 and D2–40, negative for CK and CK20. (Fig. 2g). UC and FDCS were both positive for P16, P53 and EGFR, and both negative for BRAF.

Because the second resection site was closed to the first one, we suspected the first sample might have been associated with urothelial carcinoma that was undetected in the first sample. We then obtained deeper levels of the initially resected tumor. Indeed, we identified the urothelial carcinoma in the deeper levels, which was coexisting with FDCS (Fig. 2h). After the second surgery the patient was treated with chemotherapy. At the time of writing this report, the patient had haven another relapse of urothelial carcinoma and one relapse of follicular dendritic cell sarcoma.

## Discussion and conclusions

Follicular dendritic cell sarcoma is a proliferation of spindled to ovoid-shaped neoplastic cells. There is a wide age range from 9 to 82 years associated with FDCS, with an average age of 44 years in both sexes. Men and women have similar morbidity [4]. The patient in our report was 73 years old when he first presented with urinary symptoms.

Majority of FDCS occur in cervical lymph nodes, while approximately one-third of FDCS cases occur in extranodal sites. In most cases, the patients are asymptomatic and the neoplasms usually grow slowly and painlessly. Since FDCS is rare in the bladder and has morphologic features similar to other tumors, it may create a diagnostic pitfall. FDCS may be confused with spindle cell carcinoma, malignant melanoma, lymphoma, interdigitating dendritic cell sarcoma, thymoma, and metastatic undifferentiated carcinomas, et al. But FDCS immunophenotypic profile is quite specific and useful in its diagnosis. In this case, the morphologic features and immunophenotypes (CD21, CD23, CD35, D2–40 positivity) were in keeping with follicular dendritic cell sarcoma. Majority of FDCS are considered low-grade sarcoma, while tumors with larger sizes and more mitotic figures tend to have relapses. In our case, the number of mitosis was high, which might explain the tumor recurrence 6 weeks later and the tumor cells were found to infiltrate into the muscular layer. So far only one case of FDCS was reported in urinary bladder [5]. In that case of bladder FDCS, cystitis glandularis and low-grade urothelial atypia were also found in the bladder mucosa adjacent to the tumor, which suggested that FDCS might be associated with metaplasia or even possible dysplasia in the nearby bladder mucosa. Our case is probably the first report on FDCS that occurred with UC at the same time. The UC was not present in the initial level of the first TURBT sample, which might be a useful lesson for our future practice, i.e., if there is FDCS detected in the urinary bladder, the possibility of a co-existing UC should be considered and the specimen should be examined thoroughly by examining additional tissue levels.

The etiology of FDCS is not clear. Approximately 10 to 20% of FDCS cases are associated with Castleman disease. Wang et al. reported some cases of Castleman disease contained areas of follicular dendritic cell proliferation, so FDCS was hypothesized to arise from such areas [6]. Additionally, Sun et al. stated that a similar feature of Castleman disease and FDCS was the expression of epidermal growth factor receptor (EGFR) [7]. It has also been found that, based on the study completed by Cheuk et al., Epstein-Barr virus was associated with the inflammatory pseudotumor-like variant of FDCS, which can selectively involve spleen and liver, characterized by frequent presence of systemic symptoms and marked female predominance [8]. Also FDCS is associated with complex cytogenetic abnormalities. Cell cycle regulatory gene such as P16 showed alteration [9] and tumor suppressor gene P53 mutation were also found in FDCS [10]. Recently Go et al. [11] found that the BRAF pathway was contributed to the pathogenesis of histiocytic and dendritic cell neoplasms, the BRAFV600E mutation was positive in 18.5% (5 of 27) of FDCS cases.

These cytogenetic abnormalities may share mechanisms of tumor genesis with a subset of other tumor types, such as urothelial carcinoma. P16 and P53 genes were altered more prominently in patients with high-grade tumors than low-grade tumors of urothelial carcinoma, which may play significant roles in the progression of bladder cancer [12]. There were many molecular risk factors, related to poor prognosis of UC, and one of these factors was the expression of EGFR [13]. Also, Boulalas et al. found that the involvement of BRAF mutations in the development of bladder UC was infrequent [14]. In our case, both P16 and P53 protein had shown positive stains in the FDCS and UC. Also, EGFR were over expressed, but no mutation was found about BRAF (V600E). These two tumors may share some same molecular mechanism, but the exact reason needs to be further studied.

In conclusion, follicular dendritic cell sarcoma can occur with urothelial carcinoma at the same time in the bladder, and these two tumors may share a similar molecular mechanism.

## Data Availability

All data supporting the study are presented in the manuscript or available upon request.

## Abbreviations

ALK: Anaplastic lymphoma kinase
CD: Clusters of differentiation
CK: Cytokeratin
CT: Computed tomography
EGFR: Epidermal growth factor receptor
EMA: Epithelial membrane antigen
FDCS: Follicular dendritic cell sarcoma
Melan A: Melanoma marker A
MPO: Myeloperoxidase
SMA: Smooth muscle actin
TURBT: Transurethral resection of bladder tumor
UC: Urothelial carcinoma
